# Epidemiological Study of Mortality Rate from Alcohol and Illicit Drug Abuse in Iran

**Published:** 2017-10-14

**Authors:** Seyed Mohammad Sadegh Ghoreishi, Fatemeh Shahbazi, Seyed Davood Mirtorabi, Mohammad Reza Ghadirzadeh, Seyed Saeed Hashemi Nazari

**Affiliations:** ^1^ Legal Medicine Research Center, Legal Medicine Organization, Tehran, Iran; ^2^ Department of Epidemiology, School of Public Health, Shahid Beheshti University of Medical Sciences, Tehran, Iran; ^3^ Department of Addiction Studies, School of Advanced Technologies in Medicine, Tehran Medical University of Medical Sciences, Tehran, Iran; ^4^ Safety Promotion and Injury Prevention Research Center, Department of Epidemiology, School of Public Health, Shahid Beheshti University of Medical Sciences, Tehran, Iran

**Keywords:** Epidemiology, Mortality, Opiate addiction, Psychoactive drugs

## Abstract

**Background:** The estimate of mortality associated with illicit opiate use provides useful information to those directing and monitoring local, national and international policies and programs. This study investigated the epidemiology of mortality due to the illegal consumption of narcotics and psychotropic substances in the Iran to provide evidence-based public health data for useful programs and actions aimed at preventing drug-related mortality.

**Study Design:** A cross-sectional study.

**Methods:** The information regarding all cases of psychotropic positive was collected from Legal Medicine Organization, occurred on Mar 2015 to Feb 2016. Demographic and epidemiological data were extracted from recorded documents. Data were then analyzed in Stata software.

**Results:** Overall, 2306 died cases from opioid or psychotropic abuse were evaluated. The mean age of the subjects was 36.07±12.61 yr, they were mostly single male, and 88.64% of them had Iranian nationality. The mortality rate from opiate and psychotropic abuse in the whole country was 38.22 per 1,000,000 population. The most common location of death was at home or in another private residence. History of overdose, suicide, hospitalization in psychiatric hospital, staying in prison and substance abuse in the family observed in some people who died from drug abuse.

**Conclusions:** Mortality rate from substance abuse is more among unmarried young men aged 30-39 yr with low education level also in self-employed. We suggest policies to prevent this person accessing and using drug.

## Introduction

Drug abuse is one of the chronic disorders^1^ that refers to the harmful or hazardous use of psychotropic substances including alcohol and illicit drugs^2^. Illicit drug use has been well established as a risk factor for adverse health outcomes including disability, morbidity and premature mortality^3-5^. Generally, morbidity and mortality rate among illicit drug users are more than non-drug users^6^. In addition, there is a strong association between substance and alcohol use disorders and suicidal ideation, suicide attempt and completed suicide^7-9^.

Mortality rate among illicit drug users is higher than the general population ^6, 10, 11^. Standardized mortality ratio was 13.2 (95% confidence interval (CI): 12.3 – 14.1). In other words, among people who have the same sex and age, mortality rate in drug addicts was 13 times higher than the general population^12^.

Iran, due to its special circumstances regarding adjacency to major centers of opiate drug production especially Afghanistan and exposure to the best and shortest transit route for opium, morphine base drugs, and heroin, has a long history of opiate drug use and combating drug abuse^13^. Drug abuse in Iran is considered as a social, psychological, familial and economic problem with a widespread calamity^14^.

In Iran, few studies have been done on the epidemiology of substance abuse, addiction, and their complications^15^. This research designed to describe geographical variation in mortality from substance abuse in thirty-one Iran provinces during Mar 2015 to Feb 2016 as well as demographic and epidemiological characteristics of those who died from drug abuse during this period.

## Methods

This research was a descriptive cross-sectional study. According to the Iranian law, all the suspicious deaths in the country should be investigated by the legal medicine organization centers. The death certificate is issued just by this organization for the suspicious deaths. Mortality due to illicit drug use is one of the definitions of suspicious death. Hence the study population was all the suspicious deaths referred to the centers of the legal medicine organization of Iran in all provinces during 12-month period (Mar 2015–Feb 2016) and the cause of death was illicit or alcohol drug use.

Totally 3003 deaths due to illicit drug abused were registered in legal medicine organization of Iran. In this study a sample of 2306 (76.8%) were evaluated. All the mortality rates are presented according to the total number of 3003 deaths, but the frequency tables and all other details are estimated in the sample size of 2306 persons.

We excluded all substance or alcohol-related suicides. Mortality from illicit drug use were classified using the international classification of disease, 10^th^ edition (ICD-10), based on the ICD-10 underlying cause of death codes T40 (poisoning by narcotics and psychodysleptics), F10 (mental and behavioral disorders due to use of alcohol), X42 (accidental poisoning by and exposure to narcotics and psychodysleptics [hallucinogens], not elsewhere classified), X62 (intentional self-poisoning by and exposure to narcotics and psychodysleptics [hallucinogens], not elsewhere) and Y12 (poisoning by and exposure to narcotic and psychodysleptics, not elsewhere classified, undetermined intent).

This study was performed in all the provinces of Iran and was approved by the Health Research Ethic Board in Legal Medicine Organization.

Cases were eligible for analysis if illicit substances and metabolites including opium, heroin, crack, cannabis, alcohol, and crystal quantitatively or qualitatively were determined to be the cause of death at the time of death. Samples for toxicological testing were obtained from various sites including urine, liver, bile and gastric contents. These samples were analyzed by screening method (including immune-chromatography and TLC) and confirmatory methods (containing High-Performance Liquid Chromatography and Gas Chromatography/Mass Spectrometry).

Two checklists were used to collect data. After the checklists were designed based on the study variables, several external and internal experts in forensic medicine reviewed and confirmed them. Content validity was determined by obtaining comments of professors, scholars, and expert in the field. For each checklist, a guideline was designed and taught to those who were responsible for gathering information.

Data regarding demographic characteristics including age, sex, marital status, occupation and educational level, as well as location of death (province), general health status before overdose, pre-hospital care, circumstances surrounding the event, history of suicide, type of substance, background of hospitalization in psychiatric hospital, beginning time of drug use and history of drug abuse in the family were collected through interviewing with friends and relatives of deceased.

Physicians that were responsible for the autopsy room in each province collected the data and sent them monthly to capital Legal Medicine Center in Tehran, Iran. Finally, the information extracted from the checklists was analyzed by Stata software ver. 14. Categorical variables are described with percentages, while continuous variables are described as means and standard deviations (SD) or median and interquartile distance. We did not have access to the exposed population to measure mortality rate in the exposed. Instead, the mortality rates were calculated by dividing the number of deaths due to opiate abuse by population of Iran in each province in 2015-2016 and are presented per 1,000,000 population.

## Results

**Figure 1:**
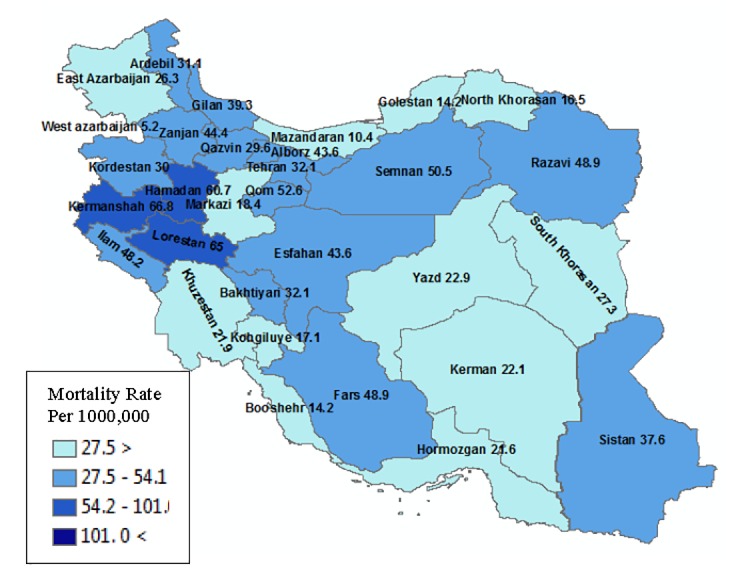
Mortality rate due to opiate and psychotropic abuse by province of residence in 12 months leading to Feb 2016 in Iran

Totally, 2306 died cases from reactions to illicit substances were enrolled. The mortality rate from substance abuse in Iran from Mar 2015 to Feb 2016 was 38.22 per 1,000,000 population. Kermanshah, Lorestan and Hamadan provinces with 66.8, 65 and 60.7 death per 1,000,000 population had the highest mortality rate respectively. Information about mortality rate that caused by drug and alcohol abuse per 1,000,000 in other provinces are depicted in Figure 1.

The mean age at death was 36.07±12.61 and the median of it was 35 years. Totally, 2052 of the cases (89.0%) were male. Most victims were Iranian (2261; 98.0%). Thirty-four deaths occurred in children who were in the age group 0-9 years. Eleven children were girl and 23 of them were boys. The distribution of the type of drug used in this age group was as follows: 10 had used methadone, two had used opium, two had used other drugs and 20 were unknown (Table 1).

**Table 1:** The absolute and relative frequency distribution of drug abuse related deaths by demographic variables in 12 months leading to Feb 2016 in Iran

**Variables**	**Number**	**Percent**
Sex		
Male	2052	89.0
Female	254	11.0
Age		
0-9	34	1.5
10-19	105	4.5
20-29	604	26.2
30-39	754	32.7
40-49	483	20.9
50-59	222	9.6
60-69	73	3.2
70-79	25	1.1
80-100	6	0.3
Nationality		
Iranian	2261	98.0
Afghan	41	1.8
Pakistani	2	0.1
Iraqi	2	0.1
Marriage status		
Single	1122	48.6
Married	875	38.0
Divorced	285	12.4
Wife died	24	1.0

The most common location of death was at home or in another private residence (1220; 52.9%). Overall, 532 patients (23%) died in hospital and 255 (11%) deaths occurred in public locations. The vast majority of deaths occurred in non-marriage individuals (1122; 48.6%).

Most of people had recognizable identity (2200; 95.4%). More than half had high school education or less (2128; 92.27%); in addition, 178 (7.73%) had attended or completed college or university. The most commonly used drug within the last one month was opium (845; 37.03%), crystal (568; 24.63%), heroin (492; 21.34%), crack (229; 9.93%), alcohol (58; 2.52%) and cannabis (43; 2.02%). Regarding the simultaneous consumption of alcohol and different illicit drugs in the last month before death, in 617 (26.75%) of cases, this history could not be obtained. In the remainder, in 60.15% cases, they used just one substance/alcohol, in 22.3% and 10.8% of them had concurrent usage of two or three substance/alcohol respectively and in 6.7% had contemporaneous usage of three or more.

Only 22.05% of subjects were living alone at time of death while most of them were not alone (1798; 77.95%). The places of their residence in one month before death are presented in Table 2. About 48.6% of deceased addicted were single, 37.6% of them were married and only 13.4% of them were divorced or widow at time of death.

**Table 2:** Distribution of history of medical conditions, war injury, location of death, employment status and Place of residence in one month before death in drug abuse related deaths in Iran in 12 months leading to Feb 2016

**Variables**	**Number**	**Percent**
History of war injury
Yes	37	1.6
No	2269	98.4
Place of residence in one month before death
Personal home	1558	67.6
Rental home	444	19.3
Revelry home	84	3.6
Addiction treatment center	7	0.3
Greenhouse	7	0.3
Hotel	5	0.2
Student dormitory	5	0.2
Prison	72	3.1
Homeless	64	2.8
Boarding house	5	0.2
Other	55	2.4
Place of death		
Home	1220	52.9
Prison	43	1.9
Addiction treatment center	51	2.2
Harm reduction center	1	0.1
Public places	255	11.0
Green house	1	0.1
Hospital	532	23
Other	203	8.8
Medical condition		
Cardio vascular disease	95	4.1
Diabetes	27	1.2
Cancers	6	0.3
Chronic pain	20	0.9
Physical disability	14	0.6
No disease/No response	2153	93.4
Employment status		
Student	38	1.6
University student	44	1.9
Housewife	173	7.5
Employee	70	3.0
Worker	269	11.6
Skilled worker	29	1.3
Solider	33	1.5
Retried	41	1.8
Unemployed	531	23.0
Farmer	35	1.5
Urban driver	51	2.2
Suburban driver	22	0.1
Military	26	1.1
Self employed	892	38.7
Drug-dealer	3	0.1
Beggar / Vendor	7	0.3
Other	40	1.7

23.24% of people died from substance abuse had history of overdose, 5.86% of them had history of suicide, and the history of detention was observed in 23.76% of them. History of hospitalization in mental hospital was also observed in 9.8% of them. Regarding the history of injection drug abuse by the victim, just 81% of families answered this question. Among them, 405 cases (24.3%) had the history of drug abuse by injection. Moreover, among 73% of families who answered this question, 28.8% had a history of drug abuse in the family.

The mean/standard deviation of age at onset of drug use among 1435 who responded to this question was 24.8±7.72 yr. The mode and median in age of starting of drug use were 20 and 24 yr respectively. Minimum age of onset of drug use was 5 yr and maximum of this was 65 yr.

The average time between onset of drug use and death by abuse was 13.7 yr with a standard deviation of 10 yr. 415 patients (18%) were under treatment at the time of death, while 1454 of them did not receive any services. The time distribution of death due to opiate and psychotropic abuse shows that most of deaths occurred in Apr (11.54%), Jul (10.41%) and Jun (9.37%).

Information about the history of medical conditions, history of war injury, employment status and place of residence in one month before death is expressed in Table 2.

**Figure 2:**
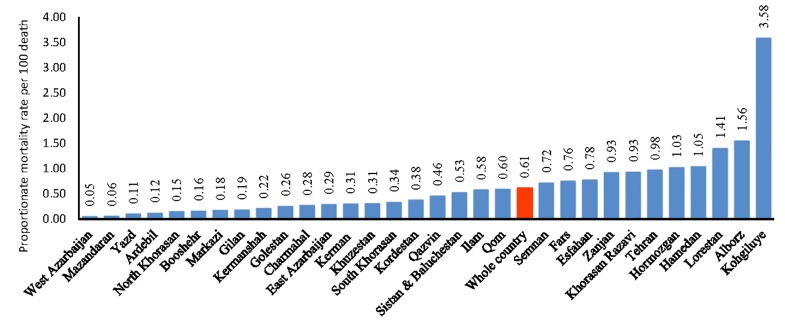
Proportionate mortality ratio from drug and psychotropic abuse in each province ordered by their rates per 100 population in 12 months leading to Feb 2016 in Iran

Figure 2 shows the proportionate mortality ratio from drug and psychotropic abuse in each province ordered by their rates per 100 population. The value of this index in Hamedan indicates, 1.05% of all deaths occurred in Hamedan Province is due to substance abuse.

**Figure 3:**
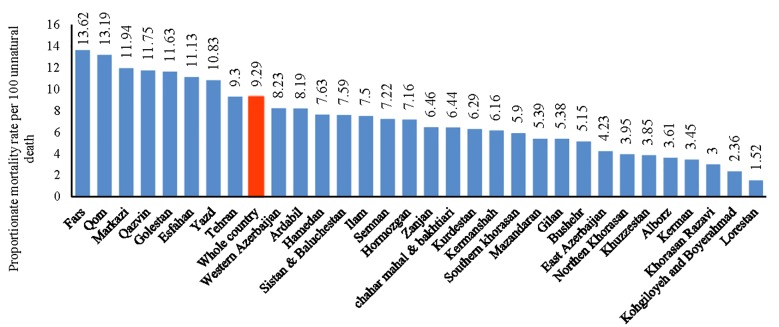
Proportionate mortality due to opiate and psychotropic abuse among unnatural causes of death in each province in 12 months leading to Feb 2016 in Iran

Death from drug abuse is one of the abnormal causes of death. Figure 3 displays the proportionate mortality due to drug abuse among unnatural causes of death in each province of Iran. Unnatural causes of death include mortality due to road traffic accidents, burning, drug abuse, drug intoxication, toxin related death, monoxide carbon (CO) intoxication, falling, firearms and cold weapons, suicide and work-related death.

## Discussion

In the present study, most of mortality due to illegal consumption of opiate substances occurs in unmarried men at the age of 30-39 yr old. Different proportions of the study population also had the history of overdose, suicide, hospitalization in psychiatric hospital, staying in prison and familial history of substance abuse.

Our finding concluded that male gender was related to mortality from substance abuse. Other studies confirm the same findings in Iran and world ^10, 13, 16, 17^. The illicit substance use was significantly higher in males than females. In the most of the societies, men have more freedom in the terms of familial and social relationships. Thus, they have more access to drugs. On the other hand, in Iranian society, women’s relationships are more controlled by their family members than men that result in fewer opportunities for illicit drug use^16^.

‏ About 38% of deaths from substance abuse occurred in married persons, inconsistent with another study in Iran ^13, 16, 18^. The pressure of family responsibility and undesired economic situations can be possible explanations, 49% of all deaths were also seen in single individuals, and so by reducing the social and moral deviations and by increasing marriage, it is possible to reduce death from drug abuse in this group.

A large proportion of the mortality from drug abuse is at the age of 30-39 yr, which is in line with other research showing how this disorder affects younger adults and lead to loss of the country’s social capital namely young persons^19-21^.

Death from opioid and psychotropic abuse is higher in people who have less education. Similar result was seen in other studies^22, 23^. By increasing the education level, the people’s awareness of adverse social and familial drug effects increases and so reduces their tendency toward drug use.

This study demonstrated that 23.76% of drug-related deaths occurred in people who had previous penal record. Previous punishments for these people were not good enough; therefore, new approaches should be followed to prevent drug abuse for these people.

Most of drug-related deaths occur in people with self-employed (38.67%) or unemployed (23.03%) ^22, 24^. Death from narcotic substance increases in people who do not have steady source of income so, an appropriate-paying vocation is essential for all young people.

Opiate-related mortality occurs in young people, who their premature mortality leads to loss of years of potential life and is responsible for enormous burden in terms of YLL (years of life lost because of premature death)^12^. Since death from opioid does not occur immediately, there is an opportunity to intervene and prevent theses death^25^. In order to enhance the effectiveness of death prevention programs, it is important to recognize circumstances around the mortality caused by opiate and psychotropic consumption. Unfortunately, inconsiderable information has been published about surrounding events and time and place of these deaths.

Based on the results of this study, the majority of opiate and psychotropic fatalities occur in home and other private places (52.9%). Therefore, family members and friends of addicts can be considered target group in the death prevention programs to prevent substance abuse overdose deaths. For example, training such individuals in overdose recognition, basic life support measures, and emergency medical services (EMS) activation may prove effective at reducing the death toll from this problem^26^.

In addition to the addict’s family circumstances, the social environment where these deaths occur is an important factor in intervention programs. In many cases, bystanders confronted with near-death events due to drug abuse do not call emergency centers or takes action to delay it, which is for the reason of fear from manslaughter charges. These behaviors lead to delays in reaching medical assistances^27, 28^.

Distrust of medical staff to chronic drug users (that usually are recognizable from their appearance) is another obstacle in providing immediate and meticulous care for these patients^29^.

Opioid overdose seems to occur in young males and in private places. Most victims do not receive bystander CPR and formal EMS services, which if activated, arrive too late for successful resuscitation to occur.

There were some limitations in our study. First, the cross-sectional nature of survey limits the ability to draw any causal inference. Second, because addiction is a sensitive issue with social stigma among population there may be some under-reporting by families.

## Conclusions

Male gender, unmarried individual, substance abuse during youth, history of narcotic abuse in the family, low education level, self-employed, unemployment, history of suicide, having a penal record, hospitalization in psychiatric hospital and injection drug use increase mortality from opiate and psychoactive abuse. We suggest policies should be taken to prevent this person accessing and using drug. Thus, the programs based on education and risk reduction strategies (for example methadone treatment) that were effective and important in supporting earlier works should be designed to reduce the mortality from drug abuse in these groups.

## Acknowledgements

The authors would like to thank Legal Medicine Organization and Legal Medicine Research Center for providing the data of this research.

## Conflict of interest statement

The authors declare that there is no conflict of interest.

## Funding

There is no source of funding.

## Highlights

- Mortality rate from drug abuse was 38.22 per 1,000,000 population.
- Opium was the most commonly used drug among the deceased.
- Mortality from drug abuse was more prevalent in unmarried young men aged 30-39 with low education level.
- The history of drug abuse in the family, hospitalization in psychiatric hospital, history of suicide and injection drug use increase mortality from drug abuse.
